# Are we moving into a new era for alcohol policy globally? An analysis of the Global Alcohol Action Plan 2022-30

**DOI:** 10.1136/bmjgh-2023-014246

**Published:** 2024-02-22

**Authors:** Jim McCambridge, Matthew Lesch

**Affiliations:** 1 Department of Health Sciences, University of York, York, UK; 2 Department of Politics and International Relations, University of York, York, UK

**Keywords:** Health policy, Public Health

## Abstract

The Global Alcohol Action Plan 2022-30 (GAAP) represents an important milestone in policy implementation at the global level on alcohol and health. There has, however, been little attention paid to the GAAP in the research literature. With a focus on the alcohol industry, this analysis examines the content of, and prospects for, the GAAP. It is clear why stronger action on alcohol and health is needed. The health harming nature of alcohol and policy interference by industry are now clearly understood. The alcohol industry is now thus regarded primarily as a key part of the problem. The GAAP calls for action in six areas with specific roles for public health actors, and invites powerful industry actors to desist from harmful activities, within each area. The broad outline of what is expected of the alcohol industry is now clear. It remains unclear, however, how far countries will continue to face formidable opposition from the major alcohol companies and their surrogates, in adopting and implementing evidence-based measures. Governments must now act at speed, and it is unclear if the targets set for 2030 will be met. If this long-running public health policy failure continues, this will have dire consequences for low and middle income countries where the alcohol market is expanding. Stronger actions may also be needed.

**SUMMARY BOX**

- Due to lack of progress on alcohol and health the 75^th^ World Health Assembly declared alcohol a public health priority and is seeking a 20% reduction in per capita consumption and other key indicators by 2030.
- The Global Alcohol Action Plan (GAAP) provides a changed view of the alcohol industry, regarding it primarily as a threat to health arising from both commercial activities and policy interference.
- The GAAP now requires alcohol companies to act ‘stringently’ within their core roles, for example in restricting marketing exposure to minors and product reformulation, and to desist from existing forms of corporate social responsibility activities.
- There are reasons for optimism and pessimism about how likely it is that GAAP will be implemented as planned, and thereby contribute to improving health worldwide.

The Global Alcohol Action Plan (GAAP), endorsed by the 75^th^ World Health Assembly (WHA) in 2022, represents an action-oriented approach to reversing growing alcohol harm.^1^ The alcohol industry, which has previously succeeded in delaying the adoption of evidence-informed policies worldwide, is expected to continue to challenge implementation of the measures included in the plan. It is therefore important to pay close attention to content on the alcohol industry within the GAAP. Eighteen months on, the WHO has not widely promoted the GAAP. In this document review we analyse GAAP’s content and prospects from a public health perspective. Our aim is to draw attention to what exactly has been agreed by the national governments of the world through the WHA, what the GAAP says about the alcohol industry, and the prospects for implementation, within the context of existing evidence.

## Why action is needed on alcohol and health

Alcohol is a widely used, intoxicating and dependence-producing toxic drug which causes approximately 3 million deaths annually.^2^ This situation is forecast to increase unless action is taken to reverse these trends.^3^ Alcohol is the only drug that is not controlled internationally by legally binding treaties.^4^

Since the 1950s, the WHO has played an important role in developing the key scientific constructs and evidence that have aided society’s attempts to address alcohol.^5^ More recently, the WHO has striven to develop a more explicit policy role, as the health, social and economic burden has grown globally.^6^ The 2010 Global Strategy for Reducing the Harmful Use of Alcohol identified 10 recommended target areas in which national governments could adopt evidence-informed interventions.^7^ Formally the GAAP was developed to ensure the effective implementation of the existing Global Strategy,^7^ which remains in force.

The WHO’s 2018 assessment found no progress had been made on the key indicator of reduced per capita consumption, and the public health imperative to strengthen efforts was clear.^4^ One observer noted that implementation has been undermined by unclear targets and underspecified goals.^6^ In response, the WHO launched the SAFER initiative in 2019 to promote the adoption of the most cost-effective but least likely implemented population-level interventions (ie, addressing alcohol pricing, availability and marketing).^8^ This represents a more focused approach, emphasising the key interventions which regulate the alcohol market more clearly. Steps were also taken through the WHO Executive Board that ultimately led the WHA to support the GAAP.^9^

## Key messages in the GAAP

The WHA has mandated a new era in alcohol policy globally, with more ambitious actions needed as part of the United Nations’ (UN) 2030 Sustainable Development Goals Agenda.^1^ This requires a more assertive posture and language than previously, to articulate and advance alcohol and public health goals. Several developments in the evidence-base permit stronger statements to be made; on the growing public health burden of alcohol-related harm, particularly in low- and middle-income countries (LMICs); on the effectiveness and cost-effectiveness of high-impact alcohol policy measures; and on the alcohol industry’s efforts to resist policy implementation.^1 2^ The GAAP states that: ‘The protection of the health of populations by preventing and reducing the harmful use of alcohol is a public health priority and should be a focus of alcohol policies and alcohol control measures implemented at different levels’ (GAAP paragraph [p] 6).^1^

The GAAP sets a goal of achieving a 20% decrease in per capita consumption, along with other targets for other indicators, by 2030. This is an increase from the 10% reduction by 2025 previously agreed in the 2013 NCD Global Monitoring Framework.1**0** Existing evidence indicates that alcohol consumption reductions of this magnitude can be expected to translate strongly into major benefits in cardiovascular disease, cancer and other cause-specific forms of mortality, and marked improvements in life expectancy.^11^ By 2030, the GAAP proposes that 70% of countries should be implementing measures to reduce affordability, availability and/or marketing. Currently, few countries are acting on all three.^9^

The GAAP identifies six key action areas for Member States, the WHO Secretariat, and international partners, civil society organisations, and academia and identifies specific actions for each. Separately, there are proposed measures for ‘economic operators’ in alcohol production and trade in each of the six areas (see box 1). These communicate clear expectations of the industry’s conduct in relation to health, including several invitations to enhance activities that are less harmful to health, and to desist from activities that are more harmful to health. Note these are invitations, not backed up by any sanctions or enforcement mechanisms.

Box 1 **Proposed measures for economic operators in alcohol production and trade in the GAAP**

**Action Area 1: Implementation of high-impact strategies and interventions**

Economic operators in alcohol production and trade are called on to focus on the implementation of measures that can contribute to reducing the harmful use of alcohol, which are stringently within their core roles as developers, producers, distributors, marketers and sellers of alcoholic beverages. They are also called on to abstain from interfering with alcohol policy development and refrain from activities that might prevent, delay or stop the development, enactment, implementation and enforcement of high-impact strategies and interventions to reduce the harmful use of alcohol.

**Action Area 2: Advocacy, awareness and commitment**

Economic operators in alcohol production and trade, as well as operators in other relevant sectors of the economy, are invited to strengthen their commitment and contribution to reducing the harmful use of alcohol within their core roles and to take concrete steps towards eliminating the marketing and advertising of alcoholic products to minors and, where relevant, towards developing and enforcing self-regulatory measures on marketing and advertising in conjunction with the development and enforcement of statutory regulations or within a co-regulatory framework. The economic operators are invited to refrain from promoting drinking; eliminate and prevent any positive health claims related to alcohol; and ensure, within regulatory or co-regulatory frameworks, the availability of easily understood consumer information on the labels of alcoholic beverages (including composition, age limits, health warnings and contraindications for alcohol consumption).

**Action Area 3: Partnership, dialogue and coordination**

Economic operators are invited to substitute, whenever possible, higher-alcohol products with no-alcohol and lower-alcohol products in their overall product portfolios, with the goal of decreasing the overall levels of alcohol consumption in populations and consumer groups, while avoiding the circumvention of existing regulations for alcoholic beverages and the targeting of new consumer groups with alcohol marketing, advertising and promotional activities. Economic operators in alcohol production and trade, as well as economic operators in other relevant sectors (such as retail, advertisements, hospitality, tourism, social media and communication), are encouraged to contribute to the elimination of marketing and sales of alcoholic beverages to minors and to the elimination of commercial activities targeted towards other high-risk groups, as well as to implement self-regulatory measures and take other actions to contribute to the elimination of such marketing practices within regulatory and co-regulatory frameworks with a legislative basis.

**Action Area 4: Technical support and capacity-building**

Economic operators in alcohol production and trade are invited to implement capacity-building activities for reducing the harmful use of alcohol within their core roles and sectors of alcohol production, distribution and sales, and to refrain from engagement in capacity-building activities outside their core roles that may undermine or compete with the activities of the public health community.

**Action Area 5: Knowledge production and information systems**

Economic operators in alcohol production and trade are called on to disclose, with due regard for the limitations associated with the confidentiality of commercial information, data of public health relevance, including a description of the methodology used to generate such data, in order to contribute to the improvement of WHO estimates of alcohol consumption in populations. This includes data on the production and sales of alcoholic beverages, as well as data on consumer knowledge, attitudes and preferences regarding alcoholic beverages.

**Action Area 6: Resource mobilisation**

Economic operators in alcohol production and trade are invited to allocate resources for the implementation of measures that can contribute to reducing the harmful use of alcohol within their core roles as developers, producers, distributors, marketers and sellers of alcoholic beverages; to refrain from funding public health and policy-related activities and research to prevent any potential bias in agenda-setting emerging from the conflict of interest; and to cease the sponsorship of scientific research on the public health dimensions of alcohol consumption and alcohol policies and its use for marketing or lobbying purposes.

The GAAP proposes that Member States: ‘Ensure that the development, implementation and evaluation of alcohol policy measures are based on public health goals and the best available evidence and are protected from the interference of commercial interests’ (Member States Area 1, Action 4 [MS, a1, a4]).^1^ Further, the WHO Secretariat are required to act on the: ‘timely countering of widespread myths and disinformation about the health effects of alcohol consumption and alcohol control measures’ (WHO Secretariat (WS) a2, a7).^1^

## No ordinary stakeholder

Economic operators in alcohol production and trade are now to be regarded as a stakeholder unlike any other, primarily as a key part of the problem not the solution. One of the GAAP’s operational principles is that: ‘The development of public policies to reduce the harmful use of alcohol should be protected, in accordance with national laws, from commercial and other vested interests that can interfere with and undermine public health objectives’ (p35).^1^

The reasons for this are set out in GAAP paragraphs 11–15 inclusive,^1^ reflecting experience globally of substantial challenges in implementing the Global Strategy, specifically including the influence of powerful commercial interests in policymaking. The alcohol industry is increasingly structured like the tobacco industry, with a small number of beer and spirits companies dominant globally.^12^ There remain important connections between the tobacco and alcohol sectors in ownership and political strategy, as there have been for many decades.^13–16^ Like tobacco, the opposition to the implementation of effective policy measures arises because of an ‘inherent contradiction between the interests of alcohol producers and public health’ (p14) that is now understood as representing ‘policy interference’ by transnational corporations and commercial interests (p14/15).^1^ One study of the alcohol industry’s tactics in the consultation processes of developing the GAAP found that industry arguments were similar to those used at national level.^17^ That study found, unlike at the national level, little evidence of successful policy interference.^17^ Further studies are warranted.

## Pinning down the alcohol industry for global health

The 2010 Global Strategy encouraged economic operators to contribute to reducing alcohol-related harm ‘within their core roles’.^7^ By contrast, the GAAP requires economic operators to act ‘stringently’ within core roles.^1^ The alcohol industry’s corporate social responsibility (CSR) activities, which often implicitly or explicitly oppose evidence-informed alcohol policies,^14 18 19^ are likely a key reason for this change in approach. GAAP implies a fundamental need to reconceptualise CSR stringently within core roles, through for example, restricting marketing exposure to minors and product reformulation to reduce alcohol content.

Among the proposed measures, Action Areas 1, 4, and 6 (see box 1) largely focus on CSR and policy interference. Past endorsements of support for the 2010 Global Strategy by major alcohol companies appear to have been primarily public relations exercises, exploiting ambiguities and weaknesses in the text, and lacking meaningful follow-through to benefits for health.^20–22^ The GAAP offers much less scope in this regard, and has outlined different accountability mechanisms. The past two decades of CSR, which is unrelated to the alcohol industry’s core business functions, is now known to have served significant policy agenda-setting functions, and strengthened lobbying capacity.^1 23^ These are important forms of policy interference, which according to GAAP should now be stopped, with monitoring of compliance needed for this and in other aspects of conduct.^1^

Production and trade interests are legitimate to have represented in policymaking. It is accepted in the GAAP, however, that alcohol policies, given the scale of the health and related societal costs, must be led by public health considerations. Policy coherence should be achieved by ensuring that health departments lead in interactions with other government departments. The proposed measures for economic operators implicitly invite compliance through co-operation with monitoring and explicitly in the provision of data of public health relevance. Monitoring is needed to assess stringency within core roles, contributions to reducing the harmful use of alcohol, and reduced activities that are harmful to public health. Interference with all aspects of alcohol policy that has the consequences of delaying the implementation of the Global Strategy and the GAAP, and in particular the high-impact strategies, should cease, and do so as soon as possible. These considerations apply to both how individual companies operate, and the alcohol industry as a whole.

Proposed measures in Action Areas 2 and 3, and to a lesser extent area 5 (see box 1) address alcohol marketing. These proposals mean very concretely taking actions such as stopping the exposure of alcohol marketing to children, eliminating misleading information, reducing the dose of ethanol within existing products, taking account of health harms in new product design and marketing, and sharing health-relevant information on request from WHO. Digital marketing is a particular challenge, as it is inherently cross-national in character, and so it presents issues for national governance requiring international co-operation. The emphasis throughout the GAAP on monitoring and evaluation reflects the action-oriented nature of the plan, and the need to be agile in capacity for response as progress is made or is not. It will also require both well-managed engagement with economic operators and vigilance regarding rapid developments in marketing. The broad outline of what society and public health expect from economic operators in this respect is now clear, and without such actions forthcoming the alcohol companies will more closely resemble the tobacco companies in rejecting what is expected of them.

## Where are we now?

The basic architecture of the 2010 Global strategy remains intact, with the key locus for decision-making and action being to develop and implement national alcohol policy. Other stakeholders now have clearly defined roles for supporting and facilitating policy action. Despite this, 18 months on from the formal adoption by the WHA, there are few indications that the GAAP has invigorated major policy innovations from Member States, or even activities by other stakeholders for whom actions have been proposed. When such efforts are made, countries can anticipate facing formidable opposition from the major alcohol companies and their surrogates, unless the GAAP itself induces change in industry approaches to policy. Where alcohol industry actors have been the key opposition and source of delay in implementing meaningful policy changes, the importance of political leadership and the mobilisation of civil society actors is clear.^24 25^

This analysis has principally focused on one major global-level development and the alcohol industry as a key impediment to progress. In analysing the prospects for the GAAP, it is essential to consider the implications of policy interference over time, as well as the challenging institutional nature of alcohol policymaking. Alcohol is a messy policy issue, which has been known for decades to be the subject of inter-departmental conflicts of interest.^26^ Not only are there many government departments involved, some are larger and more powerful than health, and may act in the interests of industry. This is partially why progress at the national level has been slow or non-existent. The GAAP’s implementation thus requires health departments to be much more assertive in promoting health interests. This will be essential, but not enough by itself, in overcoming inter-departmental tensions that may be stoked by industry actors. Governments as a whole must decide to act, and act quickly, to fulfil the commitments they have made. That requires prioritisation and political leadership. There are moot points to be made about how far this will be successful, how long it will take, and whether there will be the urgency required to meet the 2030 targets.

The alcohol industry’s contest with public health interests at the national level across the world is now being played out in a new context. What is at stake is now much clearer for all to see. The nature and significance of this long-running public health policy failure, and the reasons for it, are now more widely understood. The evidence has been available for almost half a century on which actions are needed.^2 27 28^ Alcohol is now regarded as a public health priority with a moment of opportunity that it has been deemed imperative to take. The stakes are higher. At the global level, failure to reach the 2030 target will mean that millions more will be condemned to die unnecessarily because the mandate of the governments of the world acting together in WHA has not been respected by the major players in a powerful globalised industry.

The GAAP should be seen as a starting point for policy action and not a detailed guide for arrival at a final destination. Failure to make progress will be dire in particular for those LMICs where the alcohol market is expanding.^12 29 30^ These countries are more vulnerable, lacking health and other key resources to cope. The GAAP, and progress on alcohol’s health burden, thus deserve much wider attention. Public health actors need to consider carefully how the envelope containing the mandate of the WHA can be pushed to make substantial gains for alcohol and public health.

Progress is contingent on commensurate resources being invested, without which it would be unwise to expect major gains. This may be the weakest aspect of the GAAP. The lack of high-level promotion generating widespread understanding of the need for action itself foretells that the ambitious targets may not be met. This cannot be allowed to pass. Hard questions remain to be answered about whether and how the ‘polluter pays’ principle can be applied to this issue.^31^ Innovative financing mechanisms need to be developed with real urgency. If the GAAP approach works, and that in no small part depends on what we in public health do, it can be developed further. If it does not, pressure will grow quickly for stronger measures,^32^ perhaps from as soon as the interim assessment in 2025. The activity of the research community internationally, in closely scrutinising what does or does not happen next at the national level in particular, will be important.

## Data Availability

There are no data in this work.
